# Topiramate and Metformin Are Effective Add-On Treatments in Controlling Antipsychotic-Induced Weight Gain: A Systematic Review and Network Meta-Analysis

**DOI:** 10.3389/fphar.2018.01393

**Published:** 2018-11-28

**Authors:** Chuanjun Zhuo, Yong Xu, Sha Liu, Jing Li, Qishi Zheng, Xiangyang Gao, Shen Li, Rixing Jing, Xueqin Song, Weihua Yue, Chunhua Zhou, Rachel Upthegrove

**Affiliations:** ^1^Department of Psychiatry and Morbidity, Tianjin Anding Hospital, Tianjin Medical University, Tianjin, China; ^2^Department of Psychiatry, Jining Medical University, Jining, China; ^3^Department of Psychiatry, Tianjin Medical University, Tianjin, China; ^4^Department of Psychiatry, First Hospital/First Clinical Medical College of Shanxi Medical University, Taiyuan, China; ^5^MDT Center for Cognitive Impairment and Sleep Disorders, First Hospital of Shanxi Medical University, Taiyuan, China; ^6^Department of Neurobiology, National Key Disciplines, Key Laboratory for Cellular Physiology of Ministry of Education, Shanxi Medical University, Taiyuan, China; ^7^Department of Epidemiology, Singapore Clinical Research Institute, Singapore, Singapore; ^8^Health Management Institute, Big Data Analysis Center, Chinese PLA General Hospital, Beijing, China; ^9^Department of Pattern Recognition, China National Key Laboratory, Institute of Automation, Chinese Academy of Sciences, Beijing, China; ^10^Department of Psychiatry, First Affiliated Hospital of Zhengzhou University, Zhengzhou, China; ^11^Peking University Sixth Hospital, Institute of Mental Health, Beijing, China; ^12^Key Laboratory of Mental Health, Ministry of Health & National Clinical Research Center for Mental Disorders, Peking University, Beijing, China; ^13^Department of Pharmacy, Hebei Medical University First Hospital, Shijiazhuang, China; ^14^Institute for Mental Health, University of Birmingham, Birmingham, United Kingdom; ^15^Early Intervention Service, Forward Thinking Birmingham, Birmingham, United Kingdom

**Keywords:** antipsychotic-induced weight gain, network meta-analysis, pharmacological add-ons, topiramate, metformin

## Abstract

**Background:** Antipsychotic drugs may lead to side effects such as obesity, diabetes, dyslipidemia, and cardiovascular disease. The current systematic review and network meta-analysis analyzes and provides an update on the clinical performance of these add-ons in comparison to placebo on body weight and body mass index (BMI) reductions.

**Methods:** A comprehensive literature search was performed on electronic databases: PubMed (1946-), Embase (1974-), Cochrane library (1992-), and OpenGrey (2000-) until 31 July 2018. Network meta-analyses, comparing the body weight change, BMI change and withdrawn due to adverse events of different pharmacological add-ons, was performed using a multivariate meta-regression model with random-effects, adopting a frequentist approach. To rank the prognosis for all add-ons, we used surface under the cumulative ranking (SUCRA) values.

**Outcomes:** From 614 potential studies identified, 27 eligible studies (*n* = 1,349 subjects) were included. All the studies demonstrated low to moderate risk of bias. For the analysis of body weight change, all add-ons except Ranitidine showed significant weight reductions comparing to placebo. The effectiveness rank based on SUCRA results from highest to lowest was Sibutramine, Topiramate, Metformin, Reboxetine, Ranitidine, and placebo. A similar pattern was seen for BMI change. The analysis of safety outcome did not detect significantly increased withdrawn number from the add-ons. Current evidence showed relatively good tolerance and safety of using the pharmacological add-ons.

**Interpretation:** Topiramate and Metformin are effective add-on treatments in controlling antipsychotic-induced weight gain, comparing to placebo. They are well tolerated in short-term period. Although Sibutramine has the highest rank of the effectiveness, its license has been withdrawn in many countries due to its adverse effects. Hence, Sibutramine should not be adopted to treat antipsychotic-induced weight gain.

## Introduction

Antipsychotic drugs (APDs) may lead to side effects such as obesity, diabetes, dyslipidemia, and cardiovascular disease. This adverse effect cluster presents an obstacle in the treatment and management of patients with schizophrenia or bipolar disorder, and limits patient adherence to medication and consequently adversely impacts treatment outcomes.

To counter the antipsychotic-induced weight gain, various pharmacological add-ons were investigated. Taking antidiabetics or antiobesity drugs as an adjuvant treatment, including metformin, orlistat, sibutramine, and naltrexone, is a popular approach for weight management and has been widely studied (Baptista et al., 2006; Henderson et al., 2007; McElroy et al., 2007; Joffe et al., 2008; Tchoukhine et al., 2011; Tek et al., 2014; Anagnostou et al., 2016; Rado and von Ammon Cavanaugh, 2016; Vishnupriya et al., 2016; Wu et al., 2016; Handen et al., 2017). Most of the studies reported significant reductions in body weight. Gastrointestinal agents, especially antacids like nizatidine, were reported may stop but not reduce the weight gain (Atmaca et al., 2003, 2004; Assuncao et al., 2006). Topiramate, a type of anticonvulsant, shows a negative association with body weight gain and has been found to control antipsychotic-induced weight gain for subjects with schizophrenia or bipolar disorder (McElroy et al., 2007; Afshar et al., 2009; Wozniak et al., 2009; Narula et al., 2010).

Until recently, no study has been published comparing various pharmacological add-ons on antipsychotic-induced weight gain, from both direct and indirect evidence. The current systematic review and network meta-analysis analyzes and provides an update on the clinical effectiveness and safety of these add-ons in comparison to placebo on body weight, body mass index (BMI) reductions and number of withdrawn due to adverse effects.

## Materials and Methods

### Literature Search and Eligibility Criteria

A comprehensive literature search was performed on electronic databases: PubMed (1946-), Embase (1974-), Cochrane library (1992-), and OpenGrey (2000-) until 31 July 2018. The specific concepts used in the search strategy were “antipsychotic agents” and “weight.” We conducted literature search using Medical Subject Headings (MeSH) or Emtree, and free text terms. There were no restrictions on language. The bibliography listed in review papers and included publications were also checked.

Two investigators (CjZ and QZ) independently screened for eligible studies based on pre-defined eligibility criteria. Randomized controlled trials (RCTs) that examined the pharmacological interventions of weight management for antipsychotics-induced obesity were included. To avoid imprecise estimations, only those add-ons with at least two RCTs studied were included. Non-randomized or observational studies, case reports, commentaries, and letters-to-editors were excluded.

### Data Extraction and Quality Assessment

The following data were extracted from the included studies: (1) study characteristics (publication year and patient population); (2) baseline characteristics (mean age, number of males, follow-up time, and ongoing antipsychotic treatment); and (3) outcome events (weight change [kg], BMI change [kg/m^2^], and number of withdrawn due to adverse events).

The quality of each study was evaluated, using the Cochrane Collaboration Risk of Bias tool, by two independent investigators (CjZ and QZ). Six domains were assessed for each RCT, including random sequence generation, allocation concealment, blinding of participants and personnel, blinding of outcome assessment, incomplete outcome data, selective reporting, and other sources of bias. Each domain would be assigned a judgment of ‘Low risk’ of bias, ‘High risk’ of bias, or ‘Unclear risk’ of bias. Any disagreement in quality assessment was resolved by discussion and consensus.

### Statistical Analysis

A network geometry was constructed based on the included studies for each add-on treatment. Each node represented an add-on and its size was weighted by the number of subjects of each add-on. The connecting line between two nodes meant a direct comparison existed and its thickness was determined by the number of studies included.

Network meta-analysis, comparing the body weight change, BMI change, and number of patients withdrawn due to adverse events among different pharmacological add-ons, was performed using a multivariate meta-regression model with random-effects, adopting a frequentist approach (Higgins et al., 2012; White et al., 2012). The model allows for the inclusion of potential covariates, and accounts for the correlations from multi-arm trials, and mean difference (MD) for weight and BMI change and risk ratio (RR) for number of withdrawn due to adverse events of each add-on treatment was estimated (White, 2011).

To rank the prognosis for all the add-ons, we used surface under the cumulative ranking (SUCRA) values (Salanti et al., 2011). Rank probabilities of all the add-ons were first estimated under a Bayesian framework. A step function was then applied to summarize the cumulative ranking for estimating the SUCRA values of each add-on, ranging from 0 to 1. Thus, large SUCRA values indicated a better prognosis.

The node-splitting approach and inconsistency model were used to test the consistency assumption (Dias et al., 2010). The former method involved fitting a series of node-splitting models, with one model for each add-on pairing for which there was direct and indirect evidence (Donegan et al., 2013). The latter method first fits an inconsistency model and then conduct a Wald test to check whether there is significant inconsistency among the included studies (White, 2015). Sensitivity analysis was conducted by (1) excluding studies with both “blinding of participants and personnel” and “blinding of outcome assessment” ranked as “Unclear” or “High risk,” as the outcomes (i.e., measurement of weight and BMI) were likely to be biased due to these two key components, and (2) limiting the analysis on studies with less than 12 months’ follow-up.

The network meta-analyses were implemented by Stata/MP 13 with network and network graphs package (Chaimani et al., 2013; StataCorp, 2013; White, 2015).

## Results

### Study Characteristics and Network Geometry

**FIGURE 1:**
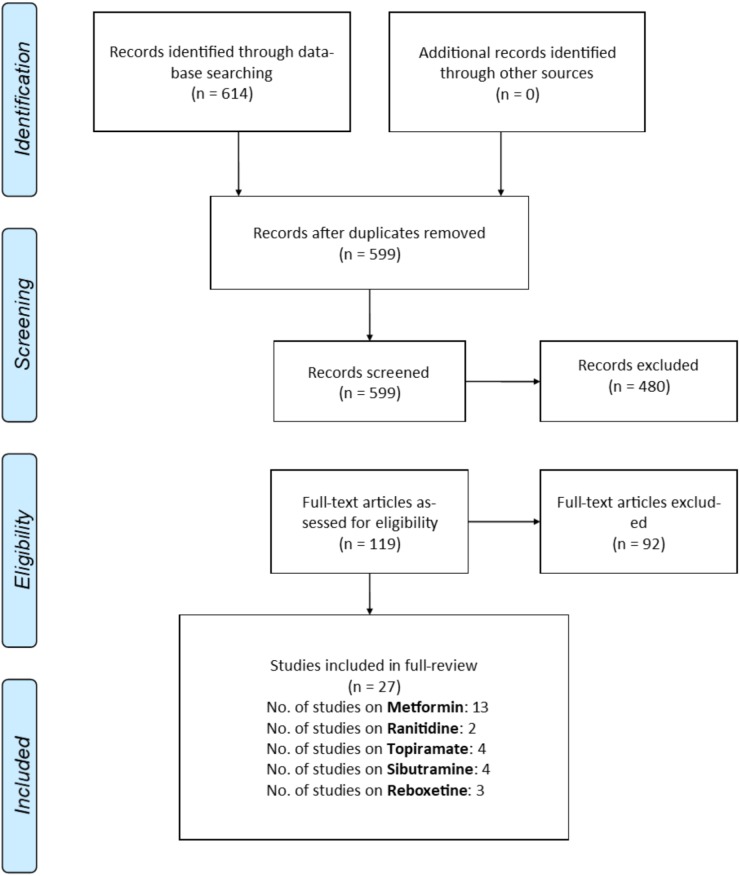
PRISMA flowchart of study selection.

From 614 potential studies identified from the initial search, 27 randomized controlled trials (*n* = 1,349 subjects) satisfied inclusion/exclusion criteria and were included in this meta-analysis (Figure 1 and Table 1; Lopez-Mato et al., 2003; Poyurovsky et al., 2003, 2007, 2013; Henderson et al., 2005, 2007; Ko et al., 2005; Nickel et al., 2005; Klein et al., 2006; Baptista et al., 2007, 2008; McElroy et al., 2007; Arman et al., 2008; Wu et al., 2008, 2012; Afshar et al., 2009; Carrizo et al., 2009; Narula et al., 2010; Wang et al., 2012; Chen et al., 2013; Jarskog et al., 2013; Ranjbar et al., 2013; Biedermann et al., 2014; de Silva et al., 2015; Anagnostou et al., 2016; Mehta and Ram, 2016; Rado and von Ammon Cavanaugh, 2016). The mean age was 31.9 years old and 48.6% (*n* = 655) were males. The follow-up period was relatively short, ranging from 6 to 26 weeks. Among the included studies, one study recruited patients with autism spectrum disorder (ASD), two for patients with bipolar disorder, 20 for patients with schizophrenia and schizophrenic conditions and four for patients with various psychosis. Efficacy results on Topiramate were reported in 4 studies, Metformin in 13 studies, Reboxetine in 3 studies, Ranitidine in 2 studies, and Sibutramine in 4 studies (Figure 1).

**Table 1:** Summary of study characteristics of included study.

Study	Country	Main diseases	Sample size	Mean age (SD)	Ongoing treatment	Intervention and control	Follow-up (weeks)
Afshar et al., 2009	Iran	Schizophrenia	I: 16 (9, 56%) C: 16 (11, 69%)	I: 37.5 (5.7) C: 38.1 (4.6)	Clo	I: Topiramate C: Placebo	8
Anagnostou et al., 2016	Canada	ASD	I: 28 (21, 75%) C: 32 (24, 75%)	I: 12.9 (2.85) C: 12.7 (2.64)	Mixed	I: Metformin C: Placebo	16
Arman et al., 2008	Iran	Schizophrenia	I: 16 (11, 69%) C: 16 (10, 63%)	I: 11.25 (2.46) C: 8.93 (4.28)	Ris	I: Metformin C: Placebo	12
Baptista et al., 2007	Canada	Schizophrenia	I: 36 (23, 64%) C: 36 (19, 53%)	I: 43.8 (11.4) C: 44.5 (12.0)	Ola	I: Metformin C: Placebo	12
Baptista et al., 2008	Canada	Schizophrenia	I: 13 (6, 46%) C: 15 (8, 53%)	I: 45.6 (8.0) C: 49.4 (12.3)	Ola	I: Metformin C: Placebo	12
Biedermann et al., 2014	Austria	Schizophrenia	I: 6 (, 0%) C: 5 (, 0%)	I: 19-65 C: 19-65	Mixed	I: Sibutramine C: Placebo	24
Carrizo et al., 2009	Venezuela	Schizophrenia	I: 24 (, 0%) C: 30 (, 0%)	I: 39.6 (9.7) C: 38.3 (8.7)	Clo	I: Metformin C: Placebo	14
Chen et al., 2013	Taiwan	Schizophrenia	I: 28 (13, 46%) C: 27 (15, 56%)	I: 41.8 (7.2) C: 41.4 (10.2)	Clo	I: Metformin C: Placebo	24
de Silva et al., 2015	Sri Lanka	Schizophrenia	I: 34 (6, 18%) C: 32 (8, 25%)	I: 33.5 (9.9) C: 35.3 (10.7)	Mixed	I: Metformin C: Placebo	26
Henderson et al., 2005	United States	Schizophrenia	I: 19 (12, 63%) C: 18 (11, 61%)	I: 43.2 (10.6) C: 40.7 (9.9)	Ola	I: Sibutramine C: Placebo	12
Henderson et al., 2007	United States	Schizophrenia	I: 11 (8, 73%) C: 10 (8, 80%)	I: 41.0 (10.0) C: 39.0 (10.0)	Clo	I: Sibutramine C: Placebo	12
Jarskog et al., 2013	United States	Schizophrenia	I: 75 (52, 69%) C: 71 (49, 69%)	I: 41.4 (11.5) C: 45.0 (10.3)	Mixed	I: Metformin C: Placebo	16
Klein et al., 2006	United States	BPD	I: 18 (9, 50%) C: 20 (12, 60%)	I: 12.9 (2.4) C: 13.3 (2.4)	Mixed	I: Metformin C: Placebo	16
Ko et al., 2005	Korea	Schizophrenia	I: 17 (7, 41%) C: 20 (12, 60%)	I: 35.3 (9.75) C: 37.6 (7.98)	Mixed	I: Topiramate C: Placebo	12
Lopez-Mato et al., 2003	Spain	Mixed	I: 29 C: 28	I: NA C: NA	Ola	I: Ranitidine C: Placebo	16
McElroy et al., 2007	United States	BPD	I: 18 (4, 22%) C: 28 (7, 25%)	I: 40.6 (13.9) C: 41.7 (11.8)	Mixed	I: Sibutramine C: Topiramate	24
Mehta and Ram, 2016	India	Schizophrenia	I: 25 (22, 88%) C: 25 (23, 92%)	I: 30.3 (7.4) C: 32.2 (8.3)	Ola	I: Ranitidine C: Placebo	8
Narula et al., 2010	India	Schizophrenia	I: 33 (22, 67%) C: 34 (22, 65%)	I: 31.2 (9.7) C: 31.0 (10.1)	Ola	I: Topiramate C: Placebo	12
Nickel et al., 2005	Germany	Mixed	I: 25 (0, 0%) C: 18 (0, 0%)	I: 35.2 (8.2) C: 34.5 (9.2)	Ola	I: Topiramate C: Placebo	10
Poyurovsky et al., 2007	Israel	Schizophrenia	I: 31 (23, 74%) C: 28 (15, 54%)	I: 30.3 (8.5) C: 29.5 (7.2)	Ola	I: Reboxetine C: Placebo	6
Poyurovsky et al., 2013	Israel	Schizophrenia	I: 29 (23, 79%) C: 14 (12, 86%)	I: 33.2 (9.7) C: 31.0 (8.2)	Ola	I: Reboxetine C: Placebo	6
Poyurovsky et al., 2003	Israel	Schizophrenia	I: 10 (6, 60%) C: 10 (5, 50%)	I: 34.6 (13.0) C: 26.5 (6.7)	Ola	I: Reboxetine C: Placebo	6
Rado and von Ammon Cavanaugh, 2016	United States	Mixed	I: 12 (7, 58%) C: 13 (5, 38%)	I: 33.5 (10.1) C: 39.08 (8.62)	Ola	I: Metformin C: Placebo	24
Ranjbar et al., 2013	Iran	Schizophrenia	I: 25 (16, 64%) C: 27 (17, 63%)	I: 38.5 (11.2) C: 37.7 (11)	Ola	I: Ranitidine C: Placebo	16
Wang et al., 2012	China	Schizophrenia	I: 32 (15, 47%) C: 34 (19, 56%)	I: 26.8 (4.2) C: 25.6 (4.6)	Mixed	I: Metformin C: Placebo	12
Wu et al., 2012	China	Schizophrenia	I: 42 (0, 0%) C: 42 (0, 0%)	I: 25.7 (4.8) C: 27.1 (4.2)	Mixed	I: Metformin C: Placebo	24
Wu et al., 2008	China	Schizophrenia	I: 18 (10, 56%) C: 19 (10, 53%)	I: 25.4 (3.9) C: 24.8 (3.5)	Ola	I: Metformin C: Placebo	12

*Clo, clozapine; Ola, olanzapine; Ris, risperidone*.

**FIGURE 2:**
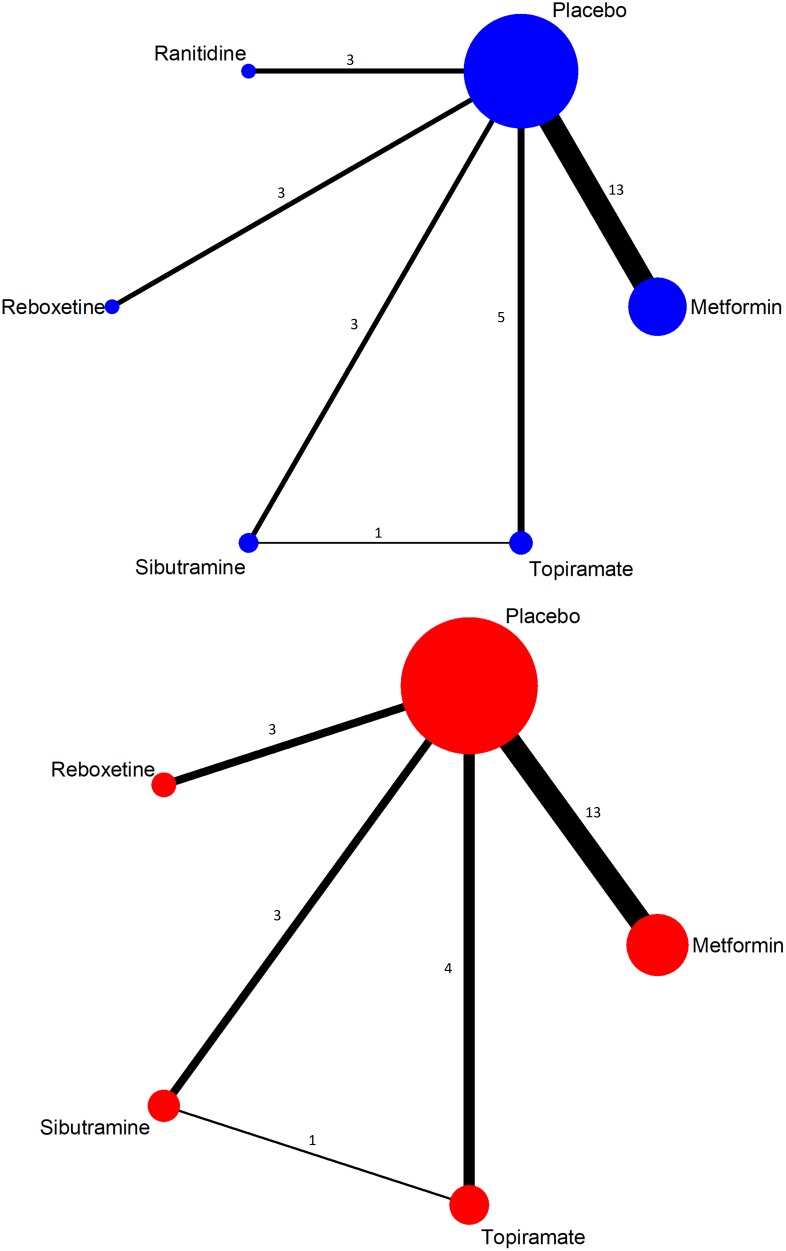
Network geometry of included studies (top, weight change; below, withdrawn due to adverse event).

The network geometry was constructed (Figure 2). Most of the studies demonstrated low to moderate risk of bias in the six domains assessed. However, due to missing information or inappropriate methods on randomization, four studies were ranked as “Unclear” or “High risk” in “Random sequence generation” (Supplementary Figure S1).

### Effectiveness on Body Weight Change

For outcome of body weight change, 27 studies were included in the analysis. All the add-ons, except Ranitidine, showed significant weight reductions compared to placebo. Topiramate showed the lowest mean difference (MD) -3.07 kg (95% CI: -5.57, -0.48), followed by Sibutramine MD = -2.97 kg (95% CI: -4.18, -1.77), Metformin MD = -2.50 kg (95% CI: -3.21, -1.80), and Reboxetine MD = -2.25 kg (95% CI: -3.54, -0.95) (Table 2). Results from both the node-splitting method and inconsistency model showed no evidence on the violation of consistency assumption between direct and indirect comparisons. As shown in Supplementary Figure S2, the pooled estimates were quite similar between consistency model (red diamonds) and inconsistency model (green diamonds), indicating that inconsistency covariates did not yield a significantly better fitting. The *p*-value = 0.166 from the Wald test further confirmed that there is no evidence on the violation of consistency assumption.

**Table 2:** Summary of results on body weight change and BMI change.

Metformin	-0.61 (-1.51, 0.30)	-0.26 (-0.93, 0.40)	0.23 (-0.78, 1.25)	0.59 (-0.51, 1.69)	-1.01 (-1.32, -0.69)^∗^
-2.40 (-4.44, -0.36)^∗^	Ranitidine	0.34 (-0.68, 1.37)	0.84 (-0.43, 2.11)	1.19 (-0.14, 2.53)	-0.40 (-1.25, 0.45)
-0.26 (-1.70, 1.18)	2.14 (-0.17, 4.46)	Reboxetine	0.50 (-0.63, 1.62)	0.85 (-0.35, 2.06)	-0.74 (-1.33, -0.16)^∗^
0.47 (-0.88, 1.83)	2.87 (0.61, 5.14)^∗^	0.73 (-1.02, 2.47)	Sibutramine	0.35 (-0.72, 1.42)	-1.24 (-2.21, -0.27)^∗^
0.57 (-2.08, 3.22)	2.97 (-0.25, 6.19)	0.83 (-2.05, 3.70)	0.10 (-2.42, 2.62)	Topiramate	-1.59 (-2.65, -0.54)^∗^
-2.50 (-3.21, -1.80)^∗^	-0.10 (-2.04, 1.83)	-2.25 (-3.54, -0.95)^∗^	-2.97 (-4.18, -1.77)^∗^	-3.07 (-5.67, -0.48)^∗^	Placebo

*Bottom left panel reported the pooled mean difference (MD) of body weight changes from network meta-analysis, and the name to the right of each MD was the reference add-on. Top right panel reported the pooled MD of BMI changes from network meta-analysis, and the name below each MD was the reference add-on; ^∗^statistical significant with *p*-value < 0.05*.

To confirm the rank of effectiveness on body weight reduction, SUCRA values were calculated, and the rank from highest to lowest was Sibutramine, Topiramate, Metformin, Reboxetine, Ranitidine, and placebo (Table 3).

In the sensitivity analysis by excluding the eight studies, similar pooled estimates were obtained and the rank order remained the same. Further sensitivity analysis by excluding studies with less than 12 months’ follow-up showed that metformin, sibutramine and topiramate were consistently significant with a reduction in body weight at -2.54 (95% CI: -3.29, -1.79), -2.98 (95% CI: -4.34, -1.62), and -2.95 (95% CI: -5.87, -0.03), respectively. Ranitidine did not show any significant reduction in body weight, which was consistent to the main result as well. However, Reboxetine was reported only in studies with less than 12 months’ follow-up, we were unable to check its sensitivity results.

### Effectiveness on BMI Change

For the BMI change outcome, 24 studies were included in the analysis. A similar pattern was seen for BMI change, where all add-ons except cardiac Ranitidine showed statistically significant BMI reductions comparing to placebo. In particular, anticonvulsant presented the highest reduction, reaching MD = -1.59 kg/m^2^ (95% CI: -2.65, -0.54) (Table 2). SUCRA results showed a consistent order from the highest to lowest: Topiramate, Sibutramine, Metformin, Reboxetine, Ranitidine, and placebo (Table 3).

### Tolerance and Safety on Number of Withdrawn Due to Adverse Events

To assess the tolerance and safety of add-on treatments, we estimated the pooled RR for all add-ons comparing to placebo. All the pooled RR had wide 95% CI due to a relatively low prevalence of patients withdrawn due to adverse events in each included study. Among them, Topiramate had the highest but statistically insignificant RR 1.88 (95% CI: 0.44, 7.94). Therefore, current evidence did not reveal a significantly higher safety issue by using add-ons in the short-term period follow-up.

**Table 3:** Summary of results from SUCRA.

Add-ons	Body weight reduction		BMI reduction		Withdrawn due to adverse event	
	SUCRA	Rank	SUCRA	Rank	SUCRA	Rank
Sibutramine	80.0	1	72.7	2	65.0	2
Topiramate	77.2	2	89.1	1	28.8	5
Metformin	64.2	3	63.5	3	34.5	4
Reboxetine	56.8	4	44.8	4	66.3	1
Ranitidine	12.4	5	26.1	5	NA	NA
Placebo	9.2	6	3.8	6	65.0	2

## Discussion

Current published systematic reviews on add-ons controlling APD-induced weight gain were either focused on one particular medication (e.g., metformin) (Housel et al., 2009; Miller, 2009; Prajapati, 2014; de Silva et al., 2016; Siskind et al., 2016), or indirectly compared different medications in a qualitative way, i.e., ranking the treatment effects simply based on the pair-wise meta-analyses results (Miller, 2009; Maayan et al., 2010; De Hert et al., 2012; Kishi et al., 2014; Mizuno et al., 2014; Choi, 2015; Whitney et al., 2015; Zimbron et al., 2016). The current review aims to quantitatively synthesize the weight and BMI reduction effects through a combination of both direct and indirect evidence. A total of 27 studies were included, among which only one study reported head-to-head comparisons between any two of the add-ons (McElroy et al., 2007). Hence, by using network meta-analysis we were able to allow indirect comparisons between the add-ons as well as against placebo.

Notably, although our results showed that Sibutramine ranked first in body weight reduction and second in BMI reduction, its license has been withdrawn in many countries due to its adverse effects. Hence, Sibutramine should not be adopted to treat antipsychotic-induced weight gain. Sibutramine is an antiobesity medication affecting both serotonin and norepinephrine reuptake (Henderson et al., 2007). Although the tolerance in this current review is not significantly lower than placebo, the Sibutramine Cardiovascular Outcomes Trial confirmed that subjects with preexisting cardiovascular disease on long-term treatment with Sibutramine had a significantly increased risk for non-fatal myocardial infarction and non-fatal stroke but not cardiovascular death or all-cause mortality (Torp-Pedersen et al., 2007). Converging with previous studies, we strongly suggest that we should not adopt sibutramine to treat antipsychotic-induced weight gain.

Some longitudinal studies suggest using body composition data rather than only measurements of body weight for understanding the relationship with disability and mortality (Seidell and Visscher, 2000; Myrskylä and Chang, 2009). Current systematic reviews included BMI changes as one of the efficacy outcomes as well. Topiramate showed the best performance on controlling BMI based on our meta-analysis. It is an anticonvulsant blocking α-amino-3-hydroxy-5-methylisoxazole-4-propionic (AMPA)/kainate-gated ion and sodium channels and positively modulate GABA receptors (White et al., 1997). The side effect of weight loss when using Topiramate has been documented and been applied to treatment for adiposity caused by eating disorders (Anghelescu et al., 2002; Levy et al., 2002). In our current review, it showed excellent weight and BMI reduction. The adverse effects, reported in the included studies, were mild to moderate without serious adverse effects. Paresthesia was the most common side effect observed in most of the included studies (Ko et al., 2005; Afshar et al., 2009; Narula et al., 2010).

Metformin, a major antidiabetic medication, was widely studied in the literature on controlling antipsychotic induced weight gain and was proven to be effective (de Silva et al., 2016; Siskind et al., 2016). Klein et al. reported its efficacy of reducing weight gain in children and adolescents who were treated with olanzapine as well (Klein et al., 2006). One of the reasons may be explained by its effects in enhancing the glycaemic control effects of insulin, antagonizing glucagon, and suppressing gluconeogenesis and glycogenolysis (Wiernsperger and Bailey, 1999). Similar reduction in BMI was reported in a recent pair-wise meta-analysis at -0.89 (95% CI -1.20, -0.58) (Zimbron et al., 2016). Although the rank of antidiabetics were generally lower than Topiramate and Sibutramine, Metformin showed long-term tolerance and weight loss effect for type 2 diabetes patients, even over 10 years (Ratner et al., 2006; Diabetes Prevention Program Research Group, 2012). This evidence highlights the potential long-term use of metformin for antipsychotic-induced weight gain patients as well.

Of note, apart from pharmacological add-ons, nutritional and behavioral interventions are important for weight control. A recent systematic review reported that non-pharmacological interventions, either individual or group interventions, or cognitive–behavioral therapy as well as nutritional counseling were effective in reducing or attenuating antipsychotic-induced weight gain compared with treatment as usual, with treatment effects maintained over follow-up (Alvarez-Jimenez et al., 2008). Our review did not consider including these interventions due to high heterogeneity from study to study, but the combination of both pharmacological and non-pharmacological interventions might be promising for further controlling the weight gain.

The current evidence in our study showed relatively good tolerance and safety of using these pharmacological add-ons. However, given that most of the included RCTs had relatively short period of follow-up, further evidence on potential long-term adverse effects are needed.

### Study Limitations

In total, only 6 out of 27 included RCTs followed up more than half a year. The relative short period of follow-up time limited the ability of our findings to be extrapolated to longer periods, as the adherence of medication, etc., may alter the effectiveness or the rank in the long run. Therefore, primary studies on the long-term effects of pharmacological add-ons are needed. As mentioned above, nutritional and behavioral intervention, which were not included in the review, are alternatives for controlling APD-induced weight gain. We would expect primary and secondary studies on evaluating the rank of effectiveness on both intervention, and additive benefits when combining both.

## Conclusion

Topiramate, and Metformin are effective add-on treatments in controlling antipsychotic-induced weight gain, comparing to placebo. They are well tolerated over a short-term period. More importantly, we propose to conduct a large sample long-term cohort study to explore the optimal treatment methods for treating antipsychotic-induced weight gain in long term.

## Ethics Statement

Ethics committee of Tianjin Anding Hospital approved this study.

## Author Contributions

CjZ, YX, ShenL, JL, QZ, and WY conceived and designed the study. QZ, WY, XS, ShaL, XG, and JL conducted the systematic review and extracted and analyzed the data. ChZ, WY, and RU drafted the manuscript. CjZ and RJ critically reviewed the manuscript for important intellectual content. All authors reviewed the manuscript. ChZ, WY, XG, and RJ had full access to all the data in the study and take the responsibility for the integrity of the data and the accuracy of the data analysis.

## Conflict of Interest Statement

The authors declare that the research was conducted in the absence of any commercial or financial relationships that could be construed as a potential conflict of interest.

## Supplementary Material

FIGURE S1 Summary of risk of bias for each study.

FIGURE S2 Forest plot of body weight change results from direct estimates, pooled with design and pooled overall (i.e., network meta-analysis).

## References

[B1] Afshar H. Roohafza H. Mousavi G. Golchin S. Toghianifar N. Sadeghi M. (2009). Topiramate add-on treatment in schizophrenia: a randomized, double-blind, placebo-controlled clinical trial. *J. Psychopharmacol.* 23 157–162. 10.1177/0269881108089816 18515465

[B2] Alvarez-Jimenez M. Hetrick S. E. Gonzalez-Blanch C. Gleeson J. F. McGorry P. D. (2008). Non-pharmacological management of antipsychotic-induced weight gain: systematic review and meta-analysis of randomized controlled trials. *Br. J. Psychiatry* 193 101–107. 10.1192/bjp.bp.107.042853 18669990

[B3] Anagnostou E. Aman M. G. Handen B. L. Sanders K. B. Shui A. Hollway J. A. (2016). Metformin for treatment of overweight induced by atypical antipsychotic medication in young people with autism spectrum disorder: a randomized clinical trial. *JAMA Psychiatry* 73 928–937. 10.1001/jamapsychiatry.2016.1232 27556593

[B4] Anghelescu I. Klawe C. Szegedi A. (2002). Add-on combination and maintenance treatment: case series of five obese patients with different eating behavior. *J. Clin. Psychopharmacol.* 22 521–524. 10.1097/00004714-200210000-00014 12352278

[B5] Arman S. Sadramely M. R. Nadi M. Koleini N. (2008). A randomized, double-blind, placebo-controlled trial of metformin treatment for weight gain associated with initiation of risperidone in children and adolescents. *Saudi Med. J.* 29 1130–1134. 18690305

[B6] Assuncao S. S. Ruschel S. I. Rosa Lde C. Campos J. A. Alves M. J. Bracco O. L. (2006). Weight gain management in patients with schizophrenia during treatment with olanzapine in association with nizatidine. *Rev. Bras. Psiquiatr.* 28 270–276. 10.1590/S1516-44462006000400005 17242805

[B7] Atmaca M. Kuloglu M. Tezcan E. Ustundag B. (2003). Nizatidine treatment and its relationship with leptin levels in patients with olanzapine-induced weight gain. *Hum. Psychopharmacol.* 18 457–461. 10.1002/hup.514 12923824

[B8] Atmaca M. Kuloglu M. Tezcan E. Ustundag B. Kilic N. (2004). Nizatidine for the treatment of patients with quetiapine-induced weight gain. *Hum. Psychopharmacol.* 19 37–40. 10.1002/hup.477 14716710

[B9] Baptista T. Martinez J. Lacruz A. Rangel N. Beaulieu S. Serrano A. (2006). Metformin for prevention of weight gain and insulin resistance with olanzapine: a double-blind placebo-controlled trial. *Can. J. Psychiatry* 51 192–196. 10.1177/070674370605100310 16618011

[B10] Baptista T. Rangel N. Fernández V. Carrizo E. El Fakih Y. Uzcátegui E. (2007). Metformin as an adjunctive treatment to control body weight and metabolic dysfunction during olanzapine administration: a multicentric, double-blind, placebo-controlled trial. *Schizophrenia Res.* 93 99–108. 10.1016/j.schres.2007.03.029 17490862

[B11] Baptista T. Uzcategui E. Rangel N. El Fakih Y. Galeazzi T. Beaulieu S. (2008). Metformin plus sibutramine for olanzapine-associated weight gain and metabolic dysfunction in schizophrenia: a 12-week double-blind, placebo-controlled pilot study. *Psychiatry Res.* 159 250–253. 10.1016/j.psychres.2008.01.011 18374423

[B12] Biedermann F. Fleischhacker W. W. Kemmler G. Ebenbichler C. F. Lechleitner M. Hofer A. (2014). Sibutramine in the treatment of antipsychotic-induced weight gain: a pilot study in patients with schizophrenia. *Int. Clin. Psychopharmacol.* 29 181–184. 10.1097/YIC.0000000000000022 24300751

[B13] Carrizo E. Fernández V. Connell L. Sandia I. Prieto D. Mogollón J. (2009). Extended release metformin for metabolic control assistance during prolonged clozapine administration: a 14 week, double-blind, parallel group, placebo-controlled study. *Schizophrenia Res.* 113 19–26. 10.1016/j.schres.2009.05.007 19515536

[B14] Chaimani A. Higgins J. P. Mavridis D. Spyridonos P. Salanti G. (2013). Graphical tools for network meta-analysis in STATA. *PLoS ONE* 8:e76654. 10.1371/journal.pone.0076654 24098547 PMC3789683

[B15] Chen C. H. Huang M. C. Kao C. F. Lin S. K. Kuo P. H. Chiu C. C. (2013). Effects of adjunctive metformin on metabolic traits in nondiabetic clozapine-treated patients with schizophrenia and the effect of metformin discontinuation on body weight: a 24-week, randomized, double-blind, placebo-controlled study. *J. Clin. Psychiatry* 74 e424–e430. 10.4088/JCP.12m08186 23759461

[B16] Choi Y. J. (2015). Efficacy of adjunctive treatments added to olanzapine or clozapine for weight control in patients with schizophrenia: a systematic review and meta-analysis. *ScientificWorldJournal* 2015:970730. 10.1155/2015/970730 25664341 PMC4310265

[B17] De Hert M. Yu W. Detraux J. Sweers K. van Winkel R. Correll C. U. (2012). Body weight and metabolic adverse effects of asenapine, iloperidone, lurasidone and paliperidone in the treatment of schizophrenia and bipolar disorder: a systematic review and exploratory meta-analysis. *CNS Drugs* 26 733–759. 10.2165/11634500-000000000-00000 22900950

[B18] de Silva V. A. Dayabandara M. Wijesundara H. Henegama T. Gunewardena H. Suraweera C. (2015). Metformin for treatment of antipsychotic-induced weight gain in a South Asian population with schizophrenia or schizoaffective disorder: a double blind, randomized, placebo controlled study. *J. Psychopharmacol.* 29 1255–1261. 10.1177/0269881115613519 26510448

[B19] de Silva V. A. Suraweera C. Ratnatunga S. S. Dayabandara M. Wanniarachchi N. Hanwella R. (2016). Metformin in prevention and treatment of antipsychotic induced weight gain: a systematic review and meta-analysis. *BMC Psychiatry* 16:341. 10.1186/s12888-016-1049-5 27716110 PMC5048618

[B20] Diabetes Prevention Program Research Group (2012). Long-term safety, tolerability, and weight loss associated with metformin in the diabetes prevention program outcomes study. *Diabetes Care* 35 731–737. 10.2337/dc11-1299 22442396 PMC3308305

[B21] Dias S. Welton N. Caldwell D. Ades A. (2010). Checking consistency in mixed treatment comparison meta-analysis. *Stat. Med.* 29 932–944. 10.1002/sim.3767 20213715

[B22] Donegan S. Williamson P. D’Alessandro U. Tudur Smith C. (2013). Assessing key assumptions of network meta-analysis: a review of methods. *Res. Synth. Methods* 4 291–323. 10.1002/jrsm.1085 26053945

[B23] Handen B. L. Anagnostou E. Aman M. G. Sanders K. B. Chan J. Hollway J. A. (2017). A randomized, placebo-controlled trial of metformin for the treatment of overweight induced by antipsychotic medication in young people with autism spectrum disorder: open-label extension. *J. Am. Acad. Child Adolesc. Psychiatry* 56 849.e–856.e. 10.1016/j.jaac.2017.07.790 28942807

[B24] Henderson D. C. Copeland P. M. Daley T. B. Borba C. P. Cather C. Nguyen D. D. (2005). A double-blind, placebo-controlled trial of sibutramine for olanzapine-associated weight gain. *Am. J. Psychiatry* 162 954–962. 10.1176/appi.ajp.162.5.954 15863798

[B25] Henderson D. C. Fan X. Copeland P. M. Borba C. P. Daley T. B. Nguyen D. D. (2007). A double-blind, placebo-controlled trial of sibutramine for clozapine-associated weight gain. *Acta Psychiatr. Scand.* 115 101–105. 10.1111/j.1600-0447.2006.00855.x 17244173

[B26] Higgins J. Jackson D. Barrett J. Lu G. Ades A. White I. (2012). Consistency and inconsistency in network meta-analysis: concepts and models for multi-arm studies. *Res. Synth. Methods* 3 98–110. 10.1002/jrsm.1044 26062084 PMC4433772

[B27] Housel A. K. Waterbury N. Argo T. R. (2009). Can metformin or rosiglitazone reduce metabolic side effects associated with atypical antipsychotics? *Issues Ment. Health Nurs.* 30 803–805. 10.3109/01612840903276712 19916816

[B28] Jarskog L. F. Hamer R. M. Catellier D. J. Stewart D. D. Lavange L. Ray N. (2013). Metformin for weight loss and metabolic control in overweight outpatients with schizophrenia and schizoaffective disorder. *Am. J. Psychiatry* 170 1032–1040. 10.1176/appi.ajp.2013.12010127 23846733 PMC3874085

[B29] Joffe G. Takala P. Tchoukhine E. Hakko H. Raidma M. Putkonen H. (2008). Orlistat in clozapine- or olanzapine-treated patients with overweight or obesity: a 16-week randomized, double-blind, placebo-controlled trial. *J. Clin. Psychiatry* 69 706–711. 10.4088/JCP.v69n0503 18426261

[B30] Kishi T. Matsuda Y. Iwata N. (2014). Cardiometabolic risks of blonanserin and perospirone in the management of schizophrenia: a systematic review and meta-analysis of randomized controlled trials. *PLoS One* 9:e88049. 10.1371/journal.pone.0088049 24505373 PMC3913743

[B31] Klein D. J. Cottingham E. M. Sorter M. Barton B. A. Morrison J. A. (2006). A randomized, double-blind, placebo-controlled trial of metformin treatment of weight gain associated with initiation of atypical antipsychotic therapy in children and adolescents. *Am. J. Psychiatry* 163 2072–2079. 10.1176/ajp.2006.163.12.2072 17151157

[B32] Ko Y. H. Joe S. H. Jung I. K. Kim S. H. (2005). Topiramate as an adjuvant treatment with atypical antipsychotics in schizophrenic patients experiencing weight gain. *Clin. Neuropharmacol.* 28 169–175. 10.1097/01.wnf.0000172994.56028.c3 16062095

[B33] Levy E. Margolese H. C. Chouinard G. (2002). Topiramate produced weight loss following olanzapine-induced weight gain in schizophrenia. *J. Clin. Psychiatry* 63:1045. 10.4088/JCP.v63n1116a 12469686

[B34] Lopez-Mato A. Rovner J. Illa G. Vieitez A. Boullosa O. (2003). Randomized, open label study on the use of ranitidine at different doses for the management of weight gain associated with olanzapine administration. *Vertex* 14 85–96. 12883589

[B35] Maayan L. Vakhrusheva J. Correll C. U. (2010). Effectiveness of medications used to attenuate antipsychotic-related weight gain and metabolic abnormalities: a systematic review and meta-analysis. *Neuropsychopharmacology* 35 1520–1530. 10.1038/npp.2010.21 20336059 PMC3055458

[B36] McElroy S. L. Frye M. A. Altshuler L. L. Suppes T. Hellemann G. Black D. (2007). A 24-week, randomized, controlled trial of adjunctive sibutramine versus topiramate in the treatment of weight gain in overweight or obese patients with bipolar disorders. *Bipolar Disord.* 9 426–434. 10.1111/j.1399-5618.2007.00488.x 17547588

[B37] Mehta V. S. Ram D. (2016). Efficacy of ranitidine in olanzapine-induced weight gain: a dose-response study. *Early Interv. Psychiatry* 10 522–527. 10.1111/eip.12205 25529756

[B38] Miller L. J. (2009). Management of atypical antipsychotic drug-induced weight gain: focus on metformin. *Pharmacotherapy* 29 725–735. 10.1592/phco.29.6.725 19476423

[B39] Mizuno Y. Suzuki T. Nakagawa A. Yoshida K. Mimura M. Fleischhacker W. W. (2014). Pharmacological strategies to counteract antipsychotic-induced weight gain and metabolic adverse effects in schizophrenia: a systematic review and meta-analysis. *Schizophr. Bull.* 40 1385–1403. 10.1093/schbul/sbu030 24636967 PMC4193713

[B40] Myrskylä M. Chang V. W. (2009). Initial BMI, weight change, and mortality among middle-and older-aged adults. *Epidemiology* 20:840. 10.1097/EDE.0b013e3181b5f520 19806061 PMC2903861

[B41] Narula P. K. Rehan H. S. Unni K. E. Gupta N. (2010). Topiramate for prevention of olanzapine associated weight gain and metabolic dysfunction in schizophrenia: a double-blind, placebo-controlled trial. *Schizophr. Res.* 118 218–223. 10.1016/j.schres.2010.02.001 20207521

[B42] Nickel M. K. Nickel C. Muehlbacher M. Leiberich P. K. Kaplan P. Lahmann C. (2005). Influence of topiramate on olanzapine-related adiposity in women: a random, double-blind, placebo-controlled study. *J. Clin. Psychopharmacol.* 25 211–217. 10.1097/01.jcp.0000162806.46453.38 15876898

[B43] Poyurovsky M. Fuchs C. Pashinian A. Levi A. Faragian S. Maayan R. (2007). Attenuating effect of reboxetine on appetite and weight gain in olanzapine-treated schizophrenia patients: a double-blind placebo-controlled study. *Psychopharmacology* 192 441–448. 10.1007/s00213-007-0731-1 17310385

[B44] Poyurovsky M. Fuchs C. Pashinian A. Levi A. Weizman R. Weizman A. (2013). Reducing antipsychotic-induced weight gain in schizophrenia: a double-blind placebo-controlled study of reboxetine-betahistine combination. *Psychopharmacology* 226 615–622. 10.1007/s00213-012-2935-2 23239133

[B45] Poyurovsky M. Isaacs I. Fuchs C. Schneidman M. Faragian S. Weizman R. (2003). Attenuation of olanzapine-induced weight gain with reboxetine in patients with schizophrenia: a double-blind, placebo-controlled study. *Am. J. Psychiatry* 160 297–302. 10.1176/appi.ajp.160.2.297 12562576

[B46] Prajapati A. R. (2014). Role of metformin in the management of antipsychotic-induced weight gain. *Prog. Neurol. Psychiatry* 18 33–38. 10.1002/pnp.358 28463344

[B47] Rado J. von Ammon Cavanaugh S. (2016). A naturalistic randomized placebo-controlled trial of extended-release metformin to prevent weight gain associated with olanzapine in a US community-dwelling population. *J. Clin. Psychopharmacol.* 36 163–168. 10.1097/JCP.0000000000000469 26872112

[B48] Ranjbar F. Ghanepour A. Sadeghi-Bazargani H. Asadlo M. Alizadeh A. (2013). The effect of ranitidine on olanzapine-induced weight gain. *Biomed. Res. Int.* 2013:639391. 10.1155/2013/639391 23984393 PMC3745912

[B49] Ratner R. Maggs D. Nielsen L. Stonehouse A. Poon T. Zhang B. (2006). Long-term effects of exenatide therapy over 82 weeks on glycaemic control and weight in over-weight metformin-treated patients with type 2 diabetes mellitus. *Diabetes Obes. Metab.* 8 419–428. 10.1111/j.1463-1326.2006.00589.x 16776749

[B50] Salanti G. Ades A. Ioannidis J. P. (2011). Graphical methods and numerical summaries for presenting results from multiple-treatment meta-analysis: an overview and tutorial. *J. Clin. Epidemiol.* 64 163–171. 10.1016/j.jclinepi.2010.03.016 20688472

[B51] Seidell J. C. Visscher T. L. (2000). Body weight and weight change and their health implications for the elderly. *Eur. J. Clin. Nutr.* 54 S33–S39. 10.1038/sj.ejcn.1601023 11041073

[B52] Siskind D. J. Leung J. Russell A. W. Wysoczanski D. Kisely S. (2016). Metformin for clozapine associated obesity: a systematic review and meta-analysis. *PLoS One* 11:e0156208. 10.1371/journal.pone.0156208 27304831 PMC4909277

[B53] StataCorp (2013). *Stata Statistical Software: Release 13*. College Station, TX: StataCorp LP.

[B54] Tchoukhine E. Takala P. Hakko H. Raidma M. Putkonen H. Rasanen P. (2011). Orlistat in clozapine- or olanzapine-treated patients with overweight or obesity: a 16-week open-label extension phase and both phases of a randomized controlled trial. *J. Clin. Psychiatry* 72 326–330. 10.4088/JCP.09m05283yel 20816037

[B55] Tek C. Ratliff J. Reutenauer E. Ganguli R. O’Malley S. S. (2014). A randomized, double-blind, placebo-controlled pilot study of naltrexone to counteract antipsychotic-associated weight gain: proof of concept. *J. Clin. Psychopharmacol.* 34 608–612. 10.1097/JCP.0000000000000192 25102328 PMC4149840

[B56] Torp-Pedersen C. Caterson I. Coutinho W. Finer N. Van Gaal L. Maggioni A. (2007). Cardiovascular responses to weight management and sibutramine in high-risk subjects: an analysis from the SCOUT trial. *Eur. Heart J.* 28 2915–2923. 10.1093/eurheartj/ehm217 17595194

[B57] Vishnupriya R. Ezhilramya J. Meenakshi B. (2016). Metformin in the prevention of metabolic syndrome associated with initiation of atypical antipsychotic therapy in adolescents and young adults-a randomized, open labeled, single centered study. *Int. J. Pharmacy Pharm. Sci.* 8 200–206.

[B58] Wang M. Tong J. H. Zhu G. Liang G. M. Yan H. F. Wang X. Z. (2012). Metformin for treatment of antipsychotic-induced weight gain: a randomized, placebo-controlled study. *Schizophr. Res.* 138 54–57. 10.1016/j.schres.2012.02.021 22398127

[B59] White H. S. Brown S. D. Woodhead J. H. Skeen G. A. Wolf H. H. (1997). Topiramate enhances GABA-mediated chloride flux and GABA-evoked chloride currents in murine brain neurons and increases seizure threshold. *Epilepsy Res.* 28 167–179. 10.1016/S0920-1211(97)00045-4 9332882

[B60] White I. R. (2011). Multivariate random-effects meta-regression: updates to mvmeta. *Stata J.* 11 255–270.

[B61] White I. R. (2015). Network meta-analysis. *Stata J.* 15 951–985.

[B62] White I. R. Barrett J. K. Jackson D. Higgins J. (2012). Consistency and inconsistency in network meta-analysis: model estimation using multivariate meta-regression. *Res. Synth. Methods* 3 111–125. 10.1002/jrsm.1045 26062085 PMC4433771

[B63] Whitney Z. Procyshyn R. M. Fredrikson D. H. Barr A. M. (2015). Treatment of clozapine-associated weight gain: a systematic review. *Eur. J. Clin. Pharmacol.* 71 389–401. 10.1007/s00228-015-1807-1 25627831

[B64] Wiernsperger N. F. Bailey C. J. (1999). The antihyperglycaemic effect of metformin. *Drugs* 58 31–39. 10.2165/00003495-199958001-00009 10576523

[B65] Wozniak J. Mick E. Waxmonsky J. Kotarski M. Hantsoo L. Biederman J. (2009). Comparison of open-label, 8-week trials of olanzapine monotherapy and topiramate augmentation of olanzapine for the treatment of pediatric bipolar disorder. *J. Child Adolesc. Psychopharmacol.* 19 539–545. 10.1089/cap.2009.0042 19877978

[B66] Wu R. R. Jin H. Gao K. Twamley E. W. Ou J. J. Shao P. (2012). Metformin for treatment of antipsychotic-induced amenorrhea and weight gain in women with first-episode schizophrenia: a double-blind, randomized, placebo-controlled study. *Am. J. Psychiatry* 169 813–821. 10.1176/appi.ajp.2012.11091432 22711171

[B67] Wu R. R. Zhang F. Y. Gao K. M. Ou J. J. Shao P. Jin H. (2016). Metformin treatment of antipsychotic-induced dyslipidemia: an analysis of two randomized, placebo-controlled trials. *Mol. Psychiatry* 21 1537–1544. 10.1038/mp.2015.221 26809842 PMC5078852

[B68] Wu R. R. Zhao J. P. Guo X. F. He Y. Q. Fang M. S. Guo W. B. (2008). Metformin addition attenuates olanzapine-induced weight gain in drug-naive first-episode schizophrenia patients: a double-blind, placebo-controlled study. *Am. J. Psychiatry* 165 352–358. 10.1176/appi.ajp.2007.07010079 18245179

[B69] Zimbron J. Khandaker G. M. Toschi C. Jones P. B. Fernandez-Egea E. (2016). A systematic review and meta-analysis of randomised controlled trials of treatments for clozapine-induced obesity and metabolic syndrome. *Eur. Neuropsychopharmacol.* 26 1353–1365. 10.1016/j.euroneuro.2016.07.010 27496573

