# Supplementary figures and images for: Topiramate and Metformin Are Effective Add-On Treatments in Controlling Antipsychotic-Induced Weight Gain: A Systematic Review and Network Meta-Analysis
Source: Front Pharmacol. 2018 Nov 28;9:1393. doi: 10.3389/fphar.2018.01393 (PMC6280187; doi:10.3389/fphar.2018.01393)

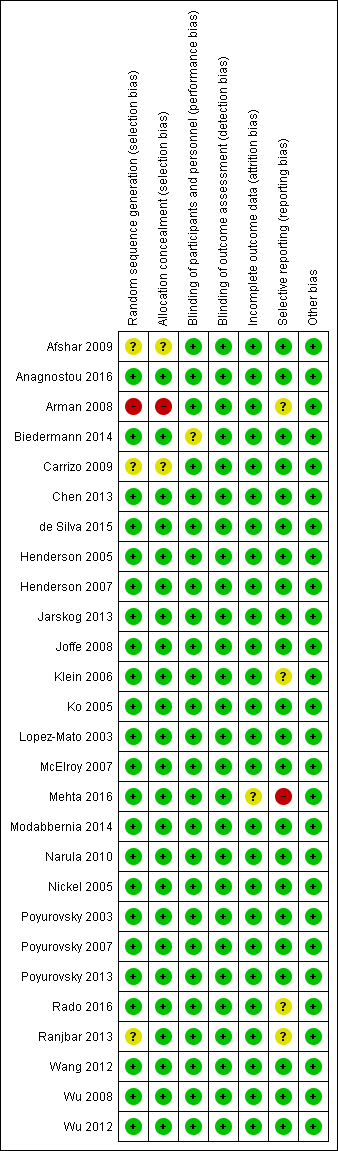

Supplement: FIGURE S1 — Summary of risk of bias for each study. [file Image_1.TIF]

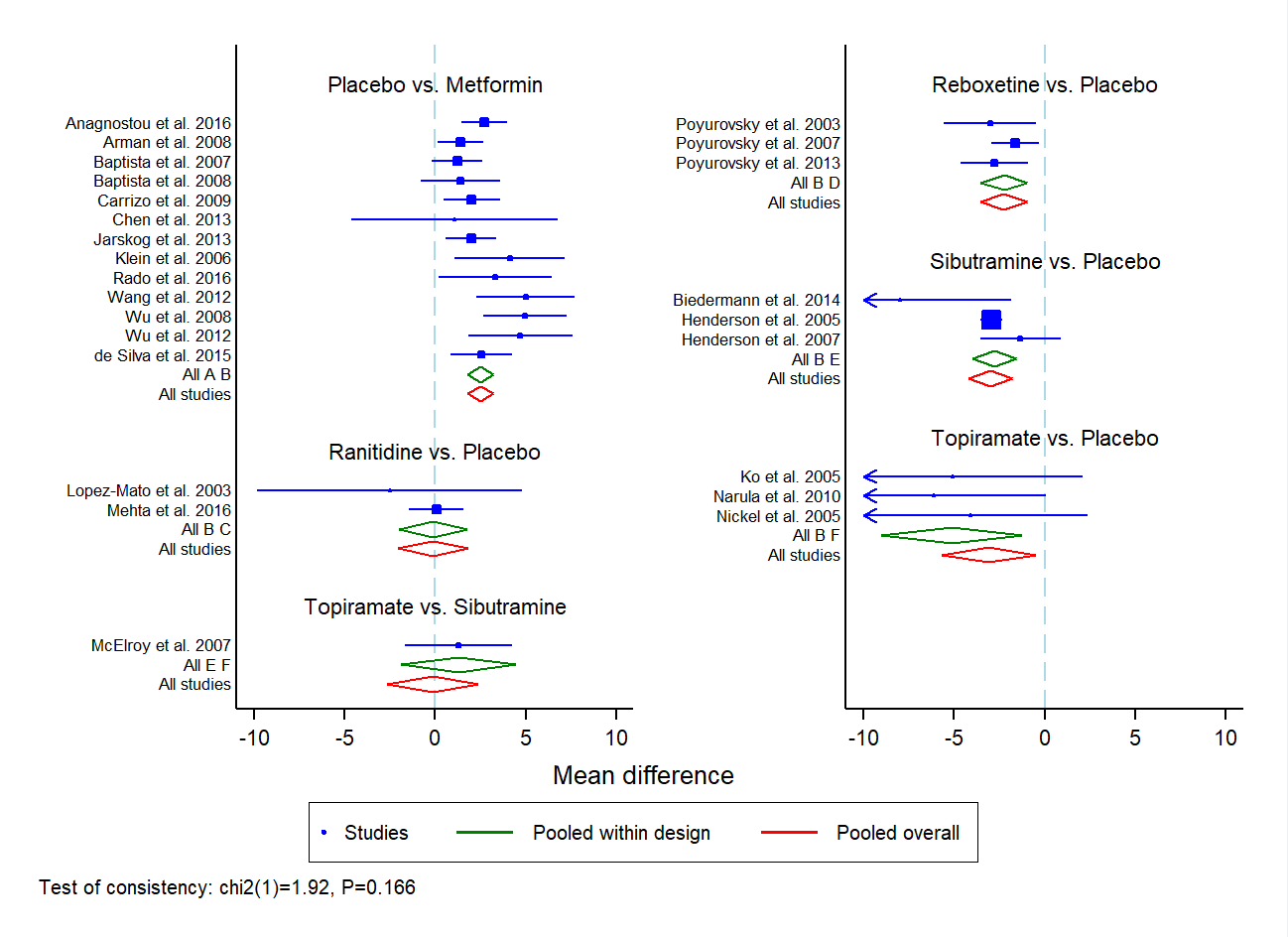

Supplement: FIGURE S2 — Forest plot of body weight change results from direct estimates, pooled with design and pooled overall (i.e., network meta-analysis). [file Image_2.TIF]
